# An mTOR and DNA-PK dual inhibitor CC-115 hinders non-small cell lung cancer cell growth

**DOI:** 10.1038/s41420-022-01082-6

**Published:** 2022-06-18

**Authors:** Fagui Chen, Huasi Zhao, Chenhui Li, Ping Li, Qichuan Zhang

**Affiliations:** 1 Department of Pulmonary and Critical Care Medicine, Shantou Central Hospital, Shantou, Guangdong China; 2 Department of Pulmonary and Critical Care Medicine, The First Affiliated Hospital of Zhengzhou University, Zhengzhou, Henan China; 3 Department of Pulmonary and Critical Care Medicine, Shenzhen People’s Hospital, Shenzhen, Guangdong China; 4 Department of Radiotherapy and Oncology, Affiliated Kunshan Hospital of Jiangsu University, Kunshan, China

**Keywords:** Non-small-cell lung cancer, Targeted therapies

## Abstract

Molecularly-targeted agents are still urgently needed for better non-small cell lung cancer (NSCLC) therapy. CC-115 is a potent DNA-dependent protein kinase (DNA-PK) and mammalian target of rapamycin (mTOR) dual blocker. We evaluated its activity in different human NSCLC cells. In various primary human NSCLC cells and A549 cells, CC-115 potently inhibited viability, cell proliferation, cell cycle progression, and hindered cell migration/invasion. Apoptosis was provoked in CC-115-stimulated NSCLC cells. The dual inhibitor, however, was unable to induce significant cytotoxic and pro-apoptotic activity in the lung epithelial cells. In primary NSCLC cells, CC-115 blocked activation of mTORC1/2 and DNA-PK. Yet, CC-115-induced primary NSCLC cell death was more potent than combined inhibition of DNA-PK plus mTOR. Further studies found that CC-115 provoked robust oxidative injury in primary NSCLC cells, which appeared independent of mTOR-DNA-PK dual blockage. In vivo studies showed that CC-115 oral administration in nude mice remarkably suppressed primary NSCLC cell xenograft growth. In CC-115-treated NSCLC xenograft tissues, mTOR-DNA-PK dual inhibition and oxidative injury were detected. Together, CC-115 potently inhibits NSCLC cell growth.

## Introduction

Lung cancer (lung carcinoma) contributes significantly to global cancer-associated mortalities [1–4]. 80–85% of all lung cancers are non‐small cell lung cancer (NSCLC) [3, 4]. In the past decades, the molecular alterations and genomic biomarkers driving lung cancer development have been explored [5–9]. For NSCLC patients with advanced diseases, targeted therapies have been applied and displayed significant benefits in prognosis and patients’ survival [5–9].

Activation of the PI3K-Akt- mTOR signaling cascade is essential for NSCLC tumorigenesis, development and progression [6, 8, 10–12]. PI3K-Akt-mTOR activation actively participates in key hallmarks of NSCLC, including sustained cancer growth, apoptosis resistance, angiogenesis, cancer invasion and metastasis and insensitivity to therapies [6, 8, 10–12]. Therefore it represents the vital therapeutic target for NSCLC [6, 8, 10–12].

The protein kinase mTOR is critical for the activation of PI3K-Akt-mTOR cascade [8, 11, 13–16]. Activated Akt will phosphorylate and inhibit tuberous sclerosis complex 2 (TSC), which then subsequently activates Rheb to activate the multi-protein complex mTORC1 (mTOR complex 1) [8, 11, 13–16]. mTORC1 then phosphorylates p70-S6 Kinase 1 (S6K) and 4EBP1, and activating transcription and translation to promote tumorigenesis [8, 11, 13–16]. mTOR complex 2 (mTORC2), that is composed of mTOR, mSin1, mLST8, Rictor, DEPTOR and several others, acts as the upstream kinase for Akt (at the Ser-473 residue) and other possible AGC kinases. When activated, mTORC2 boosts cancer progression by promoting cell proliferation and survival, cell migration and cytoskeleton remodeling [8, 11, 13–16]. Activation of both mTORC1 and mTORC2 is vital for NSCLC progression [8, 10, 11].

CC-115 is a novel and potent mTOR kinase blocker that inhibits activation of both mTORC1 and mTORC2 [17–20]. It has a favorable pharmacokinetic property. Moreover, this small molecule compound also inactivates DNA-dependent protein kinase (DNA-PK) [17–20], a high molecular weight serine/threonine kinase repairing double-strand DNA breaks via the nonhomologous end-joining mechanism [21, 22]. DNA-PK activation could promote DNA repair and offer resistance to cell death by anticancer drugs. The preclinical studies found that mTOR and DNA-PK dual inhibition by CC-115 could induce significant antitumor activity in solid tumor cells [17–20].

Zheng et al., reported that CC-115 simultaneously blocked mTOR and DNA-PK activation and inhibited renal cell carcinoma cell growth [17]. Burkel et al., reported that mTOR and DNA-PK dual inhibition by CC-115 provoked melanoma cell death and sensitized radiation-induced anti-melanoma cell activity [18]. Tsuji et al., discovered that CC-115 blocked DNA damage repair and inhibited ataxia-telangiectasia mutated kinase (ATM)-deficient cancer cell growth [20]. We here showed that targeting mTOR-DNA-PK by CC-115 remarkably hindered NSCLC cell growth.

## Results

### CC-115 induces cytotoxic, anti-proliferative and cytostatic activity in primary human NSCLC cells

**Fig. 1:**
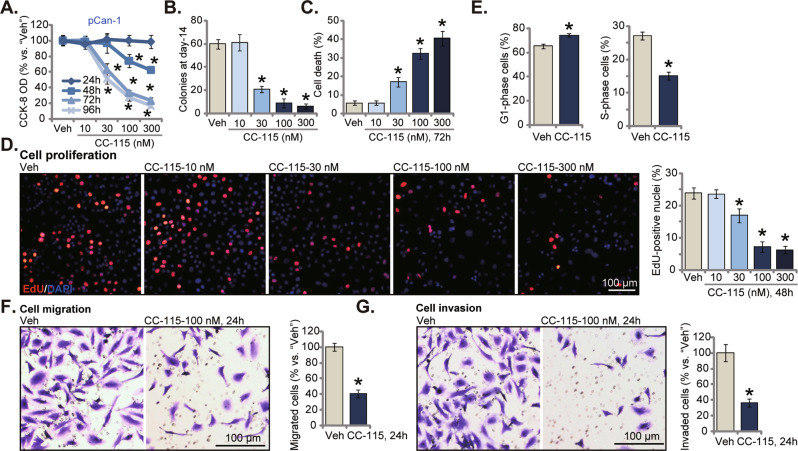
CC-115 induces cytotoxic, anti-proliferative and cytostatic activity in primary human NSCLC cells. pCan1 cells were treated with CC-115 at the applied concentrations (10–300 nM) and were cultivated for the designated time periods; Cell viability (CCK-8 OD, **A**), colony formation (colony number was quantified, **B**), cell death (by measuring Trypan blue-positive cell ratio, **C**) and proliferation (by testing nuclear EdU percentage, **D**), cell cycle progression (PI flow cytometry assays, **E**), cell migration (“Transwell” studies, **F**) and invasion (“Matrigel Transwell” studies, **G**) were tested. “Veh” stands for the vehicle control (same all for Figures). For each assay, *n* = 5. *P < 0.05 versus “Veh” group. Scale bar = 100 μm (**D**, **F** and **G**).

We first examined the potential activity of the mTOR-DNA-PK dual inhibitor in primary human NSCLC cells. The pCan1 cells [23] were treated with CC-115 (10–300 nM). CC-115 dose-dependently decreased viability (CCK-8 optical density/OD) in pCan1 cells (Fig. 1A). It was significant with 30–300 nM of CC-115 treatment (Fig. 1A). At 10 nM the dual inhibitor was ineffective and non-cytotoxic (Fig. 1A). In addition, CC-115-induced viability reduction was time-dependent (Fig. 1B). The dual inhibitor required 48 h to induce a significant cytotoxic effect in pCan-1 cells (Fig. 1A). Fig. 1B demonstrated that CC-115 (30-300 nM) remarkably inhibited viable pCan1 cell colony formation. These results further supported its cytotoxic activity. Moreover, the percentage of Trypan blue-positive staining pCan1 cells was dramatically increased after CC-115 treatment (30–300 nM, 72 h) (Fig. 1C). EdU incorporation in cell nuclei is a characteristic marker of cell proliferation. CC-115 dose-dependently decreased the EdU positively-stained nuclei ratio in pCan1 cells (Fig. 1D), suggesting proliferation inhibition. Results from these titration experiments showed that 100 nM of CC-115 treatment caused robust and significant anti-NSCLC cell activity, and this concentration was therefore chosen for following studies.

To analyze cell cycle distribution, PI flow cytometry assays were carried out. The dual inhibitor (100 nM, 36 h) resulted in G1-S arrest in pCan1 primary cells (Fig. 1E). The quantified results showed that CC-115 treatment significantly increased G1-phase cell percentage, while decreasing S-phase cell percentage in pCan1 cells (Fig. 1E). These results provided further insights to support the anti-proliferative activity by CC-115 in primary NSCLC cells. The mobility of pCan1 cells was tested as well, and “Transwell” plus “Matrigel Transwell” assays were performed. Results showed that treatment with CC-115 (100 nM, 24 h) robustly inhibited pCan1 cell migration and invasion in vitro (Fig. 1F, G). Notably, when testing cell mobility, cells were treated with the dual inhibitor for only 24 h, failing to result in significant cytotoxicity (Fig. 1A).

### CC-115 provokes apoptosis in primary human NSCLC cells

**Fig. 2:**
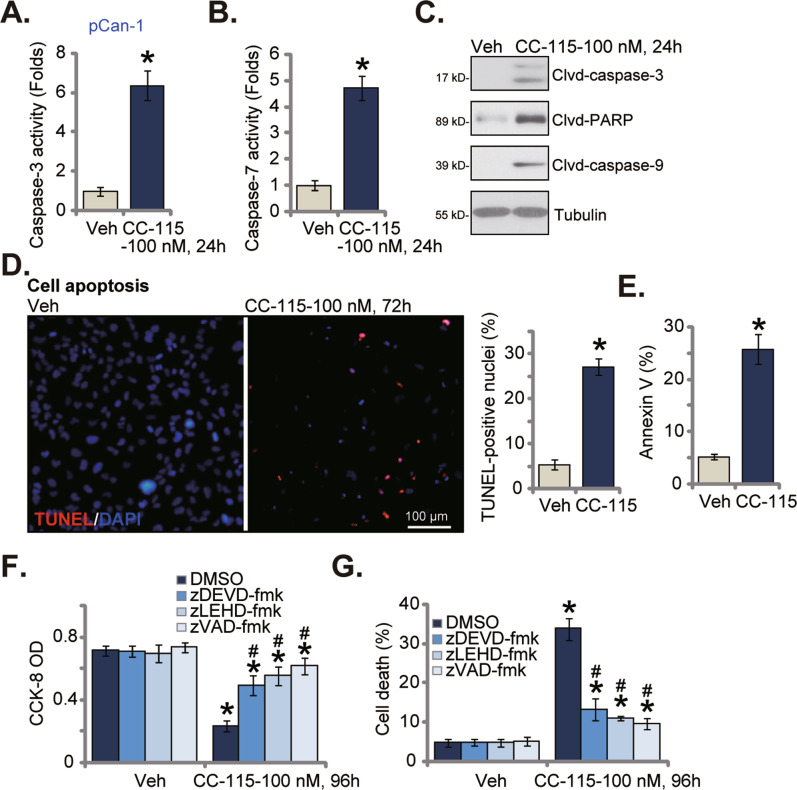
CC-115 provokes apoptosis in primary human NSCLC cells. pCan1 cells were treated with CC-115 (100 nM) and cultivated for the designated time periods, the relative caspase-3 and caspase-7 activities **A**, **B** and expression of apoptosis-related proteins **C** were examined; Cell apoptosis was measured using the nuclear TUNEL staining **D** and Annexin V flow cytometry **E** assays. pCan1 cells were pretreated for 45 min with the designated caspase inhibitors (each at 50 μM) or 0.25 % DMSO, followed by CC-115 (100 nM, 96 h) treatment, cell viability **F** and cell death **G** were examined. For each assay, *n* = 5. *P < 0.05 versus “Veh” group. ^#^*P* < 0.05 versus “DMSO” pretreatment **F**, **G**. Scale bar = 100 μm **D**.

Inhibition of mTOR can result in apoptosis in NSCLC cells [24–26]. The activities of both caspase-3 and caspase-7 were boosted in pCan1 primary cells treated with CC-115 (100 nM, 24 h) (Fig. 2A, B). Cleaved caspase-3, cleaved caspase-9 and cleaved PARP levels were remarkably boosted in CC-115-treated pCan1 cells (Fig. 2C). Figure 2D demonstrated that treatment with the dual inhibitor (at 100 nM for 72 h) robustly augmented TUNEL positively-stained nuclei ratio in pCan1 cells. In addition, the Annexin V-PI flow cytometry assay results showed that CC-115 (100 nM, 72 h) robustly increased the percentage of apoptotic pCan1 cells with Annexin V staining (Fig. 2E).

To explore the relationship between apoptosis activation and CC-115-induced cytotoxicity (see Fig. 1) in primary NSCLC cells, we utilized various caspase inhibitions, including zDEVD-fmk, zLEHD-fmk and zVAD-fmk. These caspase inhibitors largely ameliorated CC-115 (100 nM, 72 h)-induced CCK-8 viability reduction (Fig. 2F) and cell death (Fig. 2G).

### CC-115 exerts different activity in NSCLC cells and lung epithelial cells

**Fig. 3:**
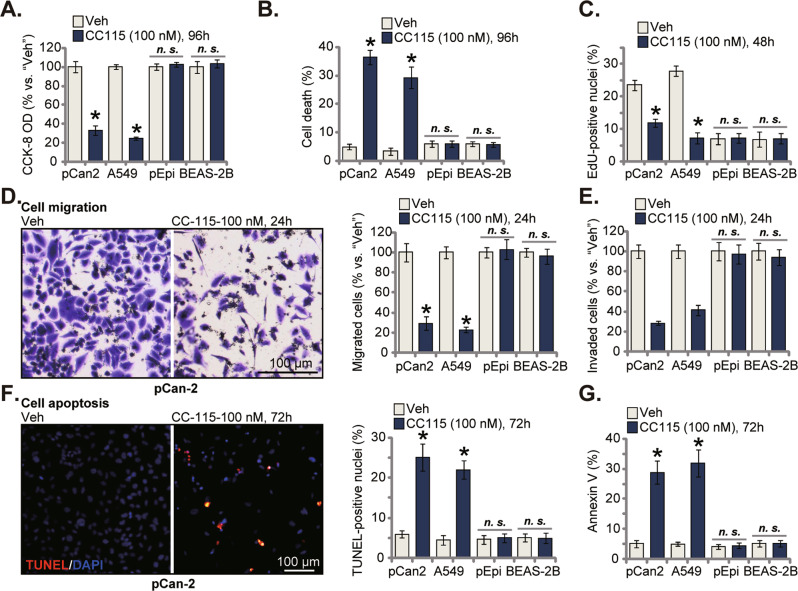
CC-115 exerts different activity in NSCLC cells and lung epithelial cells. pCan2 cells, the immortalized A549 NSCLC cells, the primary human lung epithelial cells (“pEpi”) or the BEAS-2B bronchial epithelial cells were treated with CC-115 (100 nM) and cultivated for the designated time periods; Cell viability (CCK-8 OD, **A**), cell death (**B**) and proliferation (by testing the nuclear EdU percentage, **C**), cell migration (**D**) and invasion (**E**) were tested. Cell apoptosis was measured by the nuclear TUNEL staining assay (**F**) and Annexin V flow cytometry (**G**). For each assay, n = 5. *P < 0.05 versus “Veh” group. “*n.s*.” stands for non-statistical difference. Scale bar = 100 μm (**D**).

Whether CC-115 could exert similar actions in other NSCLC cells was examined. As shown in primary NSCLC pCan2 cells and in immortalized A549 cells, treatment with CC-115 (100 nM, 96 h) remarkably inhibited CCK-8 viability (Fig. 3A) and provoked dramatic cell death (increased Trypan blue percentage, Fig. 3B). The effect of the dual inhibitor in non-cancerous lung epithelial cells was tested as well. In primary lung epithelial cells (pEpi) and the immortalized BEAS-2B bronchial epithelial cells [27], CC-115 (100 nM, 96 h) treatment however failed to induce significant viability reduction and cell death (Fig. 3A, B). Figure 3C shows that CC-115 potently inhibited proliferation of pCan2 primary cells and A549 cells, as the nuclear EdU ratio was significantly decreased (Fig. 3C). Moreover, both cell migration and invasion were inhibited by CC-115 in pCan2 and A549 NSCLC cells (Fig. 3D, E). Contrarily, treatment with the dual inhibitor failed to significantly inhibit proliferation (Fig. 3C), migration and invasion (Fig. 3D, E) in the non-cancerous pEpi and BEAS-2B cells. The TUNEL assay and Annexin V assay results showed that after CC-115 treatment robust apoptosis activation was observed only in pCan2 primary NSCLC cells and A549 cells (Fig. 3F, G). Whereas apoptosis activation was not induced by CC-115 in pEpi and BEAS-2B cells (Fig. 3F, G).

### CC-115 simultaneously blocks mTORC1/2 and DNA-PKcs activation

**Fig. 4:**
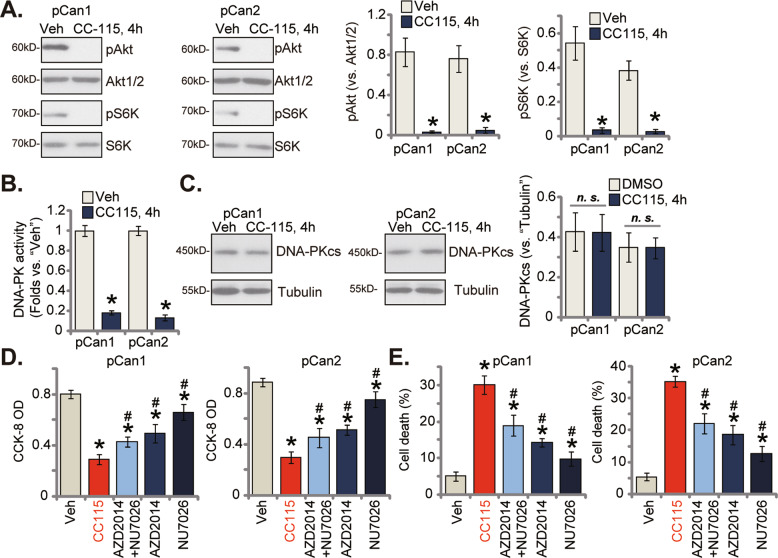
CC-115 simultaneously blocks mTORC1/2 and DNA-PKcs activation. pCan1 or pCan2 cells were treated with CC-115 (100 nM), and cultivated for the designated time periods, expression of listed proteins was shown **A**, **C**; The relative DNA-PK activity was measured as well **B**. pCan1 or pCan2 cells were treated with CC-115 (100 nM), AZD2014 (100 nM), NU7026 (100 nM) or AZD2014 plus NU7026, and cells were further cultivated for 96 h; Cell viability and death were measured via the CCK-8 **D** and the Trypan blue staining **E** assays, respectively. For each assay, *n* = 5. *P < 0.05 versus “Veh” group. ^#^*P* < 0.05 versus “CC-115” group. “*n.s*.” stands for non-statistical difference. .

CC-115 is a mTOR and DNA-PK dual blocker [20, 28, 29], we next analyzed the potential role of this compound on mTOR and DNA-PK signalings in different NSCLC cells. As shown in pCan1 and pCan2 primary NSCLC cells, treatment with CC-115 (100 nM for 4 h) almost nullified phosphorylations of Akt (at the Ser-473 residue) and S6K (at the Thr-389 residue), suggesting that CC-115, the mTOR kinase inhibitor, indeed blocked both mTORC1 and mTORC2 activation (Fig. 4A). Total Akt1/2 and S6K expression was unchanged following CC-115 treatment (Fig. 4A). Moreover, the relative DNA-PK activity was robustly decreased in CC-115-treated pCan1 and pCan2 primary cells (Fig. 4B). Contrarily, expression of DNA-PKcs was unchanged (Fig. 4C).

As shown, the mTOR kinase inhibitor AZD2014 [30] or the DNA-PK inhibitor NU7026 [31–33] induced moderate but significant cytotoxicity in primary human NSCLC cells, causing viability CCK-8 reduction (Fig. 4D) and cell death (Fig. 4E) in pCan1 and pCan2 primary cells. Yet, CC-115-induced cytotoxicity in the primary NSCLC cells was more significant than AZD2014 or NU7026 (Fig. 4D, E). Significantly, CC-115 was even more significant than AZD2014 plus NU7026 combine in inducing cytotoxicity in primary NSCLC cells (Fig. 4D, E). These results implied that mechanisms, independent of mTOR plus DNA-PK blockage, could also participate in CC-115-caused NSCLC cell death.

### CC-115 induces ROS production and oxidative injury in NSCLC cells

**Fig. 5:**
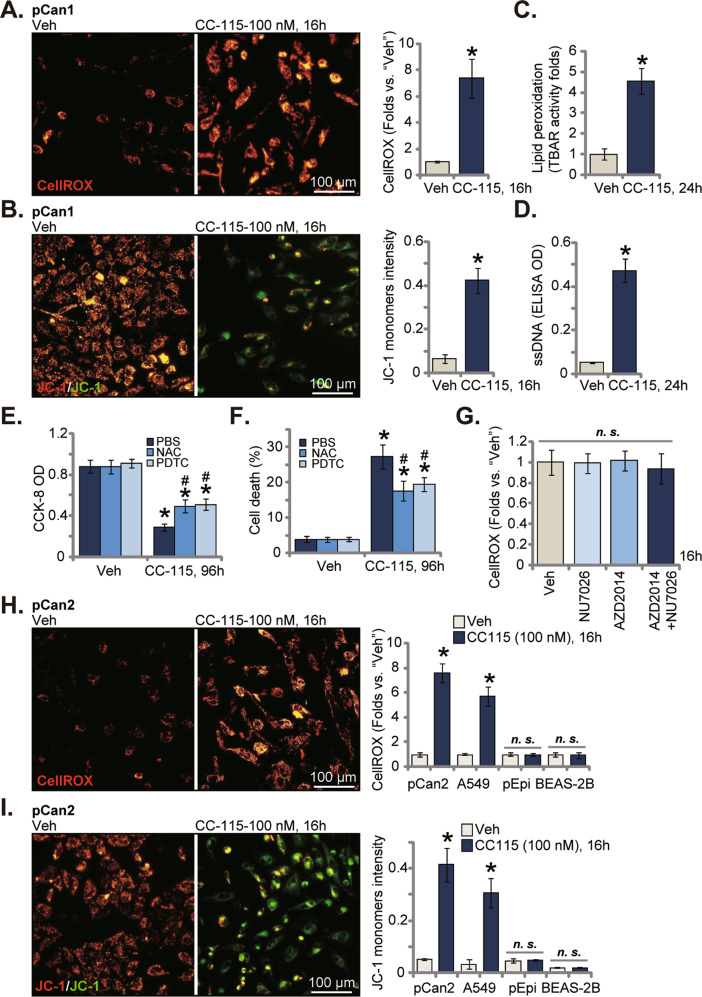
CC-115 induces ROS production and oxidative injury in NSCLC cells. The pCan1/pCan2 primary human NSCLC cells, the immortalized A549 NSCLC cells, the primary human lung epithelial cells (“pEpi”) or the BEAS-2B bronchial epithelial cells were treated with CC-115 (100 nM), and cultivated for the designated time periods; ROS production, mitochondrial depolarization, lipid peroxidation and DNA breaks were tested by measuring CellROX intensity **A**, **H**, JC-1 green monomers intensity **B**, **I**, the TBAR activity **C** and single strand DNA ELISA intensity **D**, respectively. pCan1 cells were pretreated for 35 min with n-acetyl cysteine (NAC, 500 μM), pyrrolidine dithiocarbamate (PDTC, 10 μM*)* or vehicle control (PBS), followed by CC-115 (100 nM, 96 h) treatment, cell viability **E** and death **F** were examined. pCan1 cells were treated with AZD2014 (100 nM), NU7026 (100 nM), or AZD2014 plus NU7026, cells were further cultivated for 16 h; ROS intensity was tested by measuring CellROX intensity **G**. For each assay, *n* = 5. *P < 0.05 versus “Veh” group. ^#^*P* < 0.05 versus “PBS” group **E**, **F**. “*n.s*.” stands for non-statistical difference. Scale bar = 100 μm **A**, **B**, **H**, and **I**.

Considering that CC-115-provoked NSCLC cell death was more dramatic than AZD2014 plus NU7026 combine, we tested other possible mechanisms responsible for CC-115’s actions. A number of anticancer agents, including mTOR inhibitors, can induce oxidative injury and ROS production to exacerbate cancer cell death [25, 26, 34, 35]. In CC-115 (100 nM, 16 h)-treated pCan1 primary cells, ROS levels were significantly boosted, and the CellROX intensity was robustly augmented (Fig. 5A). JC-1 transition from yellow to green (monomers) indicated mitochondrial depolarization in CC-115-stimulated pCan1 cells (Fig. 5B). Lipid peroxidation intensity was tested by analyzing TBAR activity and results showed that treatment with the dual inhibitor significantly increased lipid peroxidation in pCan1 cells (Fig. 5C). CC-115 treatment led to dramatic DNA breaks, causing ssDNA accumulation (Fig. 5D). Therefore, CC-115 induced significant oxidative injury in pCan1 cells.

Two antioxidants, NAC and PDTC, were utilized, and both ameliorated CC-115 (100 nM, 96 h)-induced CCK-8 cell viability decrease (Fig. 5E) and death (Fig. 5F) in the primary pCan1 cells. Notably, treatment with the mTOR kinase inhibitor AZD2014 and/or the DNA-PK inhibitor NU7026 was unable to induce significant ROS production in the primary pCan1 cells, and the CellROX intensity was not significantly altered (Fig. 5G). These results implied that CC-115-induced oxidative injury was likely independent of mTOR/DNA-PK inhibition. In pCan2 primary cells and immortalized A549 cells, CC-115 (100 nM) provoked ROS production (tested by the CellROX fluorescence enhancement, Fig. 5H) and depolarization of mitochondria (JC-1 yellow to green transition, Fig. 5I). Yet, the dual inhibitor failed to exert such actions in the non-cancerous pEpi cells and BEAS-2B epithelial cells (Fig. 5H, I).

### CC-115 oral administration inhibits patient-derived NSCLC xenograft growth in nude mice

At last a patient-derived xenograft model (PDX) was established by subcutaneously injecting pCan1 primary cells to the flanks of different nude mice. The pCan1 xenograft tumors were formed after three weeks after cell injection (100 mm^3^ tumor volumes, “Day-0”). The xenograft-bearing nude mice were thereafter separated randomly into two different groups. The first treatment group, containing 10 mice (*n* = 10), received daily oral administration of CC-115 (15 mg/kg). The control group mice (*n* = 10) were treated with vehicle control [17].

**Fig. 6:**
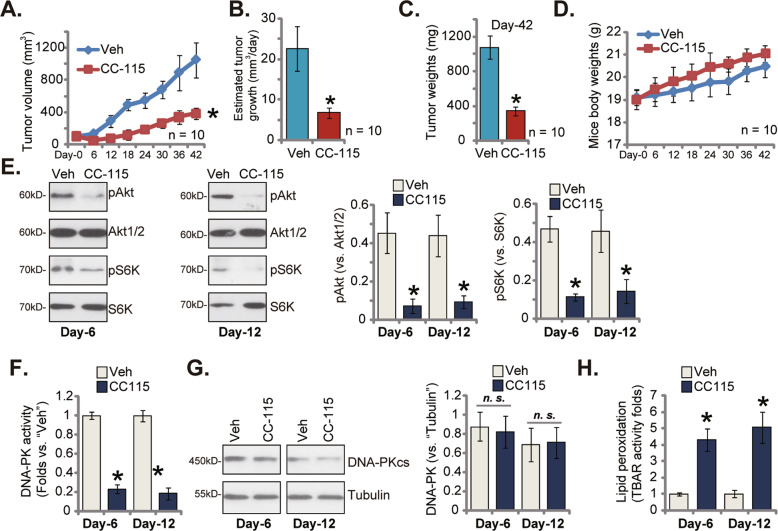
CC-115 oral administration inhibits patient-derived NSCLC xenograft growth in nude mice. The nude mice bearing pCan1 xenografts were orally administrated with a daily single dose of CC-115 (15 mg/kg) for 15 days or the vehicle control (“Veh”), the estimated tumor volumes **A** and the animal body weights **D** were recorded; The estimated daily tumor growth was measured **B**; At Day-42 tumors were isolated and weighted **C**. In the described pCan1 xenografts, expression of listed proteins was shown **E**, **G**. The relative DNA-PK activity **F** and the relative TBAR activity **H** were examined as well. For each assay, *n* = 5. *P < 0.05 versus “Veh” group.

Fig. 6A showed that oral administration of CC-115 efficiently inhibited pCan1 xenograft growth in nude mice. In CC-115-administrated mice the estimated tumor volumes were significantly lower (Fig. 6A). The daily tumor growth (presented as mm^3^ per day) was estimated using a described formula [26] and results showed that CC-115 administration dramatically suppressed pCan1 xenograft growth (Fig. 6B). At “Day-42” pCan1 xenografts were isolated and each xenograft was individually weighted. The pCan1 xenografts with CC-115 treatment were dramatically lighter (Fig. 6C). In the course of animal experiments, there was no any significant toxicities in the experimental mice. The mice body weights were indifferent between the treatment group mice and the control group mice (Fig. 6D). These results confirmed that CC-115 oral administration, at only a single dose, remarkably suppressed the growth of NSCLC xenografts in nude mice.

At Day-6/-12, one tumor of the CC-115 group and the vehicle control group was isolated (6 h after treatment), and total four xenografts were obtained. Phosphorylations of Akt and S6K were significantly inhibited in the CC-115-treated pCan1 xenograft tissues (Fig. 6E). DNA-PK activity was dramatically decreased in CC-115-treated xenograft tissues (Fig. 6F). DNA-PKcs protein expression was however unchanged (Fig. 6G). The increase of the TBAR activity indicated lipid peroxidation and oxidative injury in pCan1 xenograft tissues following CC-115 stimulation (Fig. 6H). Therefore, CC-115 inhibited mTOR and DNA-PKcs activation, and provoked oxidative injury in pCan1 xenografts.

## Discussion

A number of different mTOR inhibitors have displayed significant anti-NSCLC cell activity [6, 8, 10, 11]. Zha et al., have shown that PQR620, the mTOR kinase inhibitor, blocked mTORC1/2 and inhibited NSCLC cell growth [25]. Besides mTOR inhibition, PQR620 also induced SphK1 inactivation, ceramide accumulation and robust oxidative stress in primary NSCLC cells [25]. Yang et al., have reported that an ATP-competitive mTOR kinase inhibitor GDC-0349 impeded cell proliferation and migration, and provoked apoptosis in NSCLC cells [24]. Xia et al., reported that ASP4132, an AMPK activator, inhibited mTOR activation and suppressed NSCLC cell growth [26]. Therefore, mTOR inhibition should lead to significant anti-NSCLC cell activity.

Besides mTOR inhibition, DNA-PK inactivation could also produce dramatic anti-NSCLC activity. AZD7648, a highly-potent and specific DNA-PK inhibitor, sensitized the anti-NSCLC cell activity by radiation, chemotherapy and olaparib [36]. Pan et al., have shown that gefitinib could selectively inhibit EGFR and decrease DNA-PK activity, thereby enhancing cytotoxicity by cisplatin against NSCLC cells [37]. Liang et al., reported that DNA-PK inhibition could sensitize NSCLC cells to a third-generation EGFR blocker osimertinib [38]. In NSCLC cells osimertinib and DNA-PK inhibitor (PI-103/NU7441) together induced prolonged DNA break, cell cycle arrest and growth inhibition [38]. DNA-PK inhibition, by a small molecular inhibitor M3814, sensitized the anti-tumor activity by chemotherapeutic agents (paclitaxel and etoposide) in NSCLC cells [39]. It was shown that M3814 accelerated P53-dependent senescence response by paclitaxel and etoposide in NSCLC cells [39]. Therefore, DNA-PK is a valuable therapeutic target of NSCLC.

Here we showed that CC-115 blocked activation of both mTORC1/2 and DNA-PK, and robustly inhibited NSCLC cell growth. In various primary human NSCLC cells and A549 cells, CC-115 induced significant viability reduction, proliferation inhibition, cell cycle arrest (G1-S), and reduced in vitro cell migration. Significant NSCLC cell apoptosis was observed after CC-115 treatment. In vivo studies showed that oral administration CC-115 robustly inhibited the growth of primary NSCLC xenografts in nude mice. mTOR-DNA-PK dual inhibition and oxidative injury were detected in CC-115-treated NSCLC xenograft tissues. Intriguingly, the dual inhibitor didn’t provoke cytotoxicity non-cancerous in lung epithelial cells. Therefore, CC-115 blocks mTOR-DNA-PK activation and hinders NSCLC cell growth.

Oxidative stress and ROS enhancement were induced in NSCLC cells following treatment with a number of cytotoxic agents, thereby facilitating cell apoptosis [40–42]. Conversely, ROS-scavenging agents (NAC and others) can ameliorate NSCLC cell death by anti-cancer drugs [25, 40–42]. We here found that CC-115 provoked oxidative injury in primary NSCLC cells. CC-115-induced NSCLC cell apoptosis was ameliorated by two well-known antioxidants NAC and PDTC, indicating that oxidative injury participated in NSCLC cell apoptosis by CC-115. Importantly, we proposed that CC-115-induced oxidative stress in NSCLC cells was an unique action and was independent of mTOR-DNA-PK dual inhibition. Indeed, co-treatment with ZD2014 and NU7026 failed to provoke ROS production in primary NSCLC cells. This should further supported the superior anti-NSCLC cell activity by CC-115, more potent that ZD2014 and NU7026 combination.

## Conclusion

Novel and more efficient therapeutic options against NSCLC are urgently needed [6, 43]. We found that CC-115 potently inhibited NSCLC cell growth, representing as a promising and valuable anti-NSCLC agent.

## Materials and methods

### Chemicals, reagents and antibodies

CC-115 was from Dr. Zheng [17]. Antibodies utilized in the present study were previously described [24]. Pyrrolidine dithiocarbamate (PDTC), n-acetyl cysteine (NAC), zDEVD-fmk, zLEHD-fmk, zVAD-fmk, AZD2014 and NU7026 were purchased from Sigma (St. Louis, Mo).

### Cell culture

A549 cells and BEAS-2B epithelial cells as well as the primary human NSCLC cells, derived from two written-informed consent patients, pCan1 and pCan2, were from Dr. Shi [23]. Cells were cultured as described [23]. The protocols were approved by the Shantou Central Hospital. Cells were routinely checked and verified.

### Cellular function studies

Cellular function studies, including viability by cell counting kit-8 (CCK-8), colony formation assay, propidium iodide (PI)-flow cytometry assaying of cell cycle progression, Annexin V-PI flow cytometry assaying of cell apoptosis were described in elsewhere [44]. The protocols testing caspase-3 and caspase-7 activities were described early [45]. Other assays, including “Transwell” assaying of in vitro cell migration and invasion, the nuclear EdU (5-ethynyl-2’-deoxyuridine)-DAPI double staining of cell proliferation, Trypan blue staining, JC-1 fluorescent staining of mitochondrial depolarization, ssDNA (single-strand DNA) ELISA and TUNEL assaying of cell apoptosis were described in detail in previous studies [23, 46, 47]. The CellROX fluorescent staining assay of reactive oxygen species (ROS) intensity was reported early [24]. The detailed protocols of DNA-PK activity assay were described previously [48]. Detection of cellular and tissue lipid peroxidation intensity via the thiobarbituric acid reactive substance (TBAR) method [49] was based on the previously described protocols [50, 51].

### Protein detection

The detailed protocols of Western blotting assay and data quantification were described previously [24]. Fig. S1 showed the uncropped blotting images.

### Xenograft animal studies

Four-five week old nude mice (half male and half female, 18.5–19.5 g of weight) were maintained at the Animal Facility of Sun Yat-sen University. As reported previously [25], pCan-1 primary cells (at six million cells each mouse) were subcutaneously injected to flanks of nude mice and patient-derived xenografts (PDX) were formed within three weeks, and tumor volumes were nearly 100 mm^3^. The xenograft-bearing nude mice were separated into two random groups, receiving the applied CC-115 administration or vehicle control treatment. The latter was described previously [25]. Measuring of tumor volumes was reported previously [25]. All animal experiments were approved by Institutional Animal Care and Use Committee and Ethics Board of Shantou Central Hospital.

### Statistical analysis

No samples and animals were excluded from the analysis. The data in the present study were all with normal distribution. All data were presented as mean ± standard deviation (SD). All in vitro experiments were repeated five times, and similar results were observed. Statistical analyses were reported early [24].

## Supplementary information

Figure S1.

## Data Availability

All data are available upon request.
