# Supplementary figures and images for: An mTOR and DNA-PK dual inhibitor CC-115 hinders non-small cell lung cancer cell growth
Source: Cell Death Discov. 2022 Jun 18;8:293. doi: 10.1038/s41420-022-01082-6 (PMC9206683; doi:10.1038/s41420-022-01082-6)

Figure S1: The uncropped blotting images

Figure 2

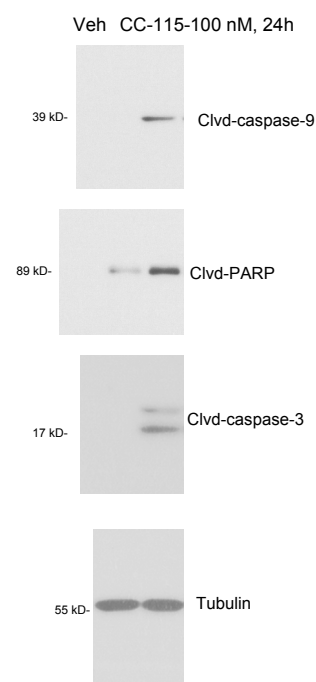

Figure 4

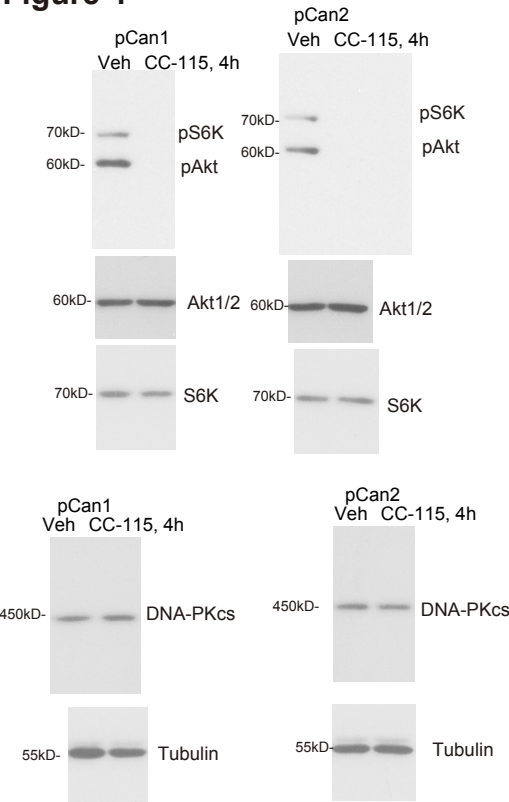

Figure 6

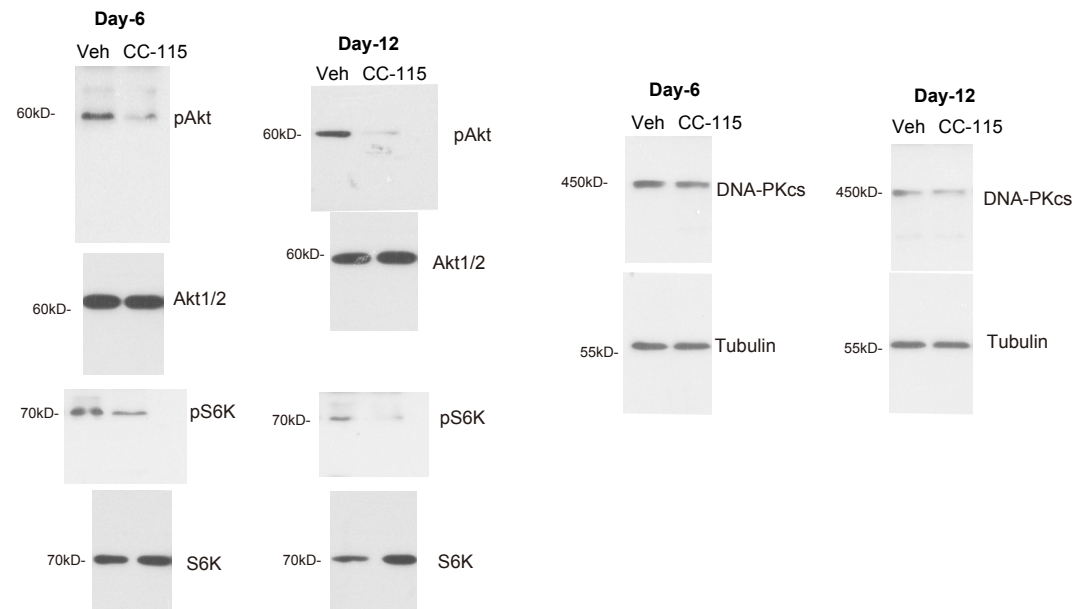

Supplement: Supplementary file 1 — Figure S1. [file 41420_2022_1082_MOESM1_ESM.pdf]
